# A systematic review of brain morphometry related to deep brain stimulation outcome in Parkinson’s disease

**DOI:** 10.1038/s41531-022-00403-x

**Published:** 2022-10-13

**Authors:** Fengting Wang, Yijie Lai, Yixin Pan, Hongyang Li, Qimin Liu, Bomin Sun

**Affiliations:** 1 Department of Neurosurgery, Ruijin Hospital, Shanghai Jiao Tong University School of Medicine, Shanghai, China; 2 Department of Psychology and Human Development, Vanderbilt University, Nashville, USA

**Keywords:** Parkinson's disease, Magnetic resonance imaging

## Abstract

While the efficacy of deep brain stimulation (DBS) is well-established in Parkinson’s Disease (PD), the benefit of DBS varies across patients. Using imaging features for outcome prediction offers potential in improving effectiveness, whereas the value of presurgical brain morphometry, derived from the routinely used imaging modality in surgical planning, remains under-explored. This review provides a comprehensive investigation of links between DBS outcomes and brain morphometry features in PD. We systematically searched PubMed and Embase databases and retrieved 793 articles, of which 25 met inclusion criteria and were reviewed in detail. A majority of studies (24/25), including 1253 of 1316 patients, focused on the outcome of DBS targeting the subthalamic nucleus (STN), while five studies included 57 patients receiving globus pallidus internus (GPi) DBS. Accumulated evidence showed that the atrophy of motor cortex and thalamus were associated with poor motor improvement, other structures such as the lateral-occipital cortex and anterior cingulate were also reported to correlated with motor outcome. Regarding non-motor outcomes, decreased volume of the hippocampus was reported to correlate with poor cognitive outcomes. Structures such as the thalamus, nucleus accumbens, and nucleus of basalis of Meynert were also reported to correlate with cognitive functions. Caudal middle frontal cortex was reported to have an impact on postsurgical psychiatric changes. Collectively, the findings of this review emphasize the utility of brain morphometry in outcome prediction of DBS for PD. Future efforts are needed to validate the findings and demonstrate the feasibility of brain morphometry in larger cohorts.

## Introduction

Deep brain stimulation (DBS) is a well-established treatment for advanced Parkinson’s Disease (PD)^1^. Despite the generally promising outcomes, the outcome of DBS varies across patients. Considering the pivotal importance of refining patient selection process and improving surgical benefits, predicting the outcome and complication of the DBS surgery in PD is necessary^2^. Progress in methodology has provided new insights into outcome prediction. Advanced imaging technologies such as brain tractography and functional MRI have been applied in the outcome prediction of DBS. These techniques share the advantages of testing a wide range of neural properties. However, they also require expertize in acquiring high-quality raw data and in deciphering the results, which may limit their utility in clinical practice^3^.

As a standard procedure used for presurgical planning^4^, structural MRI based on T1-weighted or T2-weighted sequences has its advantage in clinical practice owing to the relatively short scanning time and popularization across surgical centers^5^. Several imaging biomarkers have been proposed to predict the progression of PD including the morphometry of substantia nigra^6^. Post-processing methods, such as voxel-based morphometry (VBM) are widely applied, which help support disease diagnosis, track clinical progressions, and monitor treatment effects^7–9^.

However, recent findings on the T1-weighted imaging associated with DBS benefits revealed more discrepancies than it solved. Analyses of the motor cortex showed promising results for the prediction of motor outcomes^10,11^, whereas some study negated the associations^12^. Volumes of brain ventricles were suggested in some studies to predict postsurgical outcomes^13–16^, while other studies did not corroborate these associations^17,18^. Also, the value of these biomarkers in the prediction of DBS outcomes has not been fully investigated. It, therefore, remains to be clarified how and where presurgical MRI findings on brain morphometry can help in predicting clinical response to DBS in PD patients. This review systematically analyzes existing evidence on the association of DBS outcome and MRI findings on brain morphometry. Interpretations of associations between brain structures and surgery outcomes are discussed. Limitations in current studies are also pointed out to assist future efforts in the search for reliable imaging predictors.

## Results

### Literature search

**Fig. 1:**
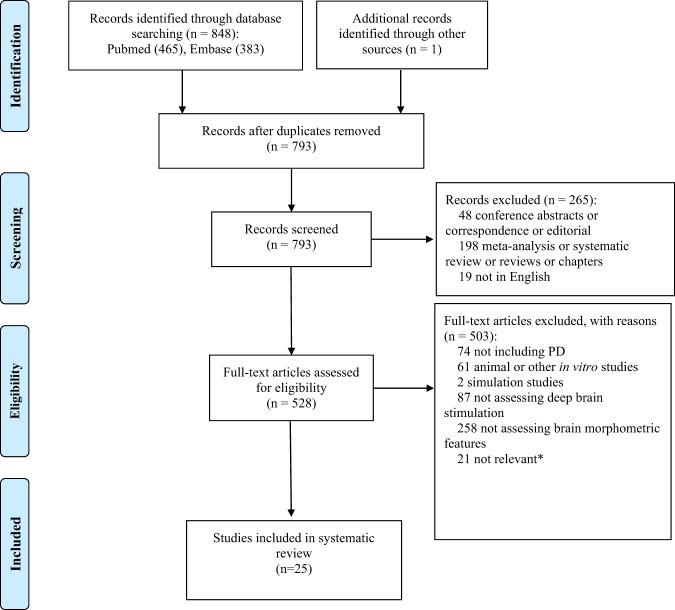
The flow diagram of the literature selection process. *Studies considered as not relevant included 11 studies focusing on the procedure of DBS targeting, 6 studies assessing postsurgical morphometry features, and 4 studies not assessing the clinical outcome.

**Fig. 2:**
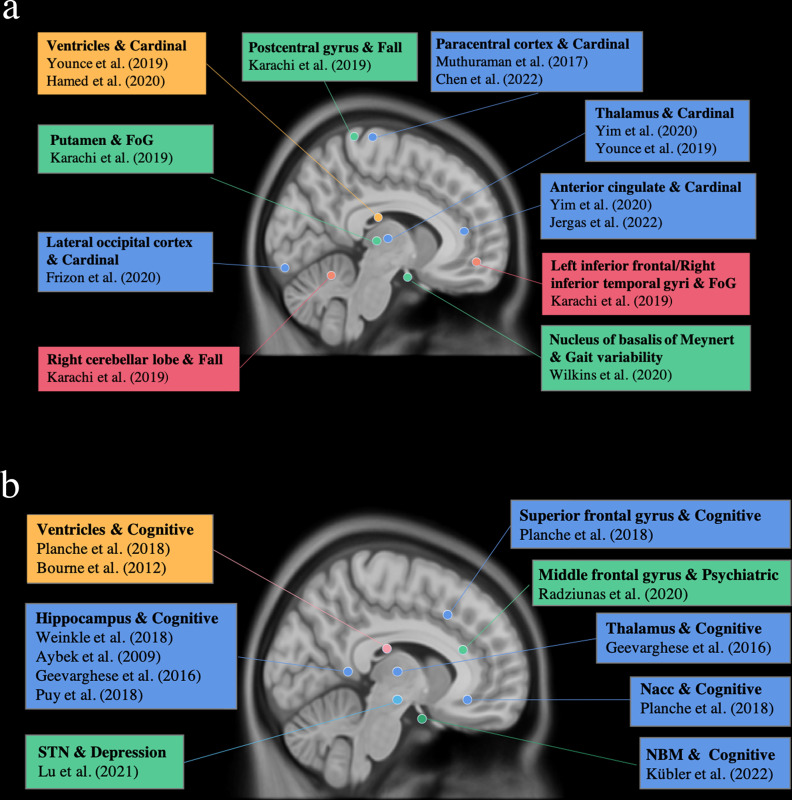
Regions associated with motor (a) and non-motor (b) outcomes. Blue and green indicate positive correlation between the regional volume/cortical thickness and postsurgical performance. Yellow and pink indicate negative correlation between the regional volume/cortical thickness and postsurgical performance.

After the removal of duplicates, 793 publications were scanned for title and abstract and a total of 25 studies were included in this review after full-text review (Fig. 1): 13 studies analyzed the motor outcome of DBS including two studies focusing on the axial symptoms (Table 1, Supplementary Table 1); and 13 studies investigated non-motor outcomes, including cognitive impairment (eight studies) and psychiatric changes (six studies) (Fig. 2).

**Table 1:** Studies investigating the associations between structural MRI features and motor outcomes in our review.

Author; year	Pts (male/female)	Duration of disease, yr	Age, yr	Associations	LFU	MDS-/UPDRS III before	MDS-/UPDRS III after
Cardinal motor symptoms							
Bonneville; 2005^21^	40 (25/15) BL STN	13.9 ± 4.7	56.6 ± 8.2	↓ Mesencephalon (nonsignificant, *p* < 0.06) & Increased UPDRSIII _med off, stim on_.	6 mo	Med off:45.8 ± 20.2; Med on:12.0 ± 11.4	Med off:16.9 ± 14.6; Med on:7.3 ± 8.7
Hamasaki; 2010^19^	21 (9/12) BL STN	11.9 ± 6.2	66.0 ± 7.9	↑ WMF & Increased percent improvement of UPDRSIII _med off/on; med off/on, stim on_.	3 mo	Med off:42.4 ± 15.0; Med on:23.9 ± 17.6	Med off:13.8 ± 11.0; Med on:11.6 ± 10.5
Price; 2011^17^	37 (28/9) 11 UL GPi; 26 UL STN	148 ± 66 mo	58.8 ± 7.0	The lateral ventricular volume did not correlate with the absolute improvement of UPDRS III _med off/med off, stim on_.	4 mo	Med off:44.2 ± 12.0	Med off:34.3 ± 10.6
Muthuraman; 2017^11^	31 (23/8) BL STN	16.0 ± 6.2	63.4 ± 9.3	↑ Cortical thickness of the frontal lobe (paracentral area and superior frontal region) & Increased UPDRSIII _med off, stim on_/ UPDRSIII _med off_.	NR, at least 3 mo after surgery	Med off:38.9 ± 11.7; Med on:18.7 ± 8.2;	Med off:19.2 ± 9.4
Younce; 2019^13^	86 (58/28) BL STN	11.8 ± 4.4	62.9 ± 9.5	↓ Ventricular volumes and ↑ thalamic volumes & Increased absolute improvement of UPDRS III _med off/med off, stim on_.	15 mo	Med off:36.7 ± 10.2	Med off:21.0 ± 7.1
Frizon; 2020^12^	36 (31/5) BL STN	9.2 ± 3.3	64.1 ± 5.6	↑ Cortical thickness of the left lateral-occipital cortex & Increased improvement of MDS-UPDRS III _med off/med off, stim on_.	6 mo	Med off:46.0 ± 15.7	Med off:21.2 ± 11.3
Hamed; 2020^14^	34 (28/6) BL GPi	10.2 ± 4.6	62.4 ± 9.2	↑Bicaudate ratio, the Evans index, and the third ventricular width & Increased UPDRSIII _med off, stim on_/ UPDRSIII _med off_.	6 mo	Med off: 52.2 ± 13.4	NR
Yim; 2020^23^	81 (40/41) STN	9.95 ± 4.6	59.0 ± 8.9	↑ Volumes of the anterior cingulate and right thalamus in the higher motor improvement (MI) group.	1 yr	Med off:50.1 ± 14.0; Med on:14.7 ± 9.8	Med off: Higher MI group:4.2 ± 3.6; Lower MI group:26.6 ± 16.9
Lu; 2021^22^	59 (33/26) 57 BL STN 2 BL GPi	8.2 ± 4.3	PD:65.7 ± 7:5; Control:59.8 ± 7.1	The left or right STN volume and ICV did not correlate with pre- and post- UPDRSIII scores.	6 mo	NR	NR
Chen; 2022^10^	Training set: 73 (45/28); Test set: 21 (11/10) BL STN	Training set:8.0 [5.9, 11.0]; Test set: 8.0[6.0, 11.9]	Training set :63.0 [57.0,68.3]; Test set:63.0 [57.0,68.3]	↑ Cortical thickness of the right precentral cortex & Increased percent improvement in MDS-UPDRSIII _med off/med off, stim on_.	4–5 weeks after surgery	Med off:Training set: 51.0 ± 17.3; Test set: 50.5 ± 16.7; Med on:Training set: 25.4 ± 15.0; Test set: 26.0 ± 14.6	Med off: Training set:26.2 ± 15.2; Test set:27.6 ± 14.1;
Jergas; 2022^20^	39 (23/16) BL STN	NR	male:62.0 ± 8.3; female:63.6 ± 5.2	↑ Volume of the frontoparietal cortex & Increased percent improvement in UPDRSIII _med off/med off, stim on_.	3 mo	Med off:38.1 ± 10.8;	Med off:25.5 ± 12.9;
Axial symptoms							
Price; 2011^17^	37 (28/9) 11 UL GPi; 26 UL STN	148 ± 66 mo	58.8 ± 7.0	The lateral ventricular volume did not correlate with the improvement of axial motor scores.	4 mo	Med off:44.2 ± 12.0	Med off:34.3 ± 10.6
Karachi; 2019^24^	331 (207/124); (151 in VBM analysis) STN	12.5 ± 5.0	57.7 ± 8.4	↓ Volume of the putamen & FOG worsening ↓ Volume of the left postcentral gyrus & Falls	1 yr	NR	NR
Wilkins; 2020^25^	23 (16/7, one excluded) BL STN	8.1 ± 3.2	59.2 ± 10.1	↓ Volume of the NBM & Increase in swing time variability (did not survive FWE correction)	63.9 ± 31.9 d, A subset (11/22) of pts completed the SIP task after 3 yr.	Med off:42.5 ± 11.9; Med on:20.2 ± 8.7	Med off:15.0 ± 8.1

*STN* subthalamic nucleus, *GPi* internal globus pallidus, *NBM* nucleus of basalis of Meynert, *BL* bilateral, *UL* unilateral, *WMF* white matter fraction, *GMF* gray matter fraction, *CSFF* cerebrospinal fluid fraction, *ICV* intracranial volume, *UPDRS* Unified Parkinson’s Disease Rating Scale, *HAM* Hamilton Depression Scale, *MMSE* Mini Mental Status Examination, *MOCA* Montreal Cognitive Assessment, *PDQ-39* 39-item Parkinson’s Disease Questionnaire scale, *MDRS* Mattis Dementia Rating Scale, *FDR* false discovery rate, *FWE* family-wise error corrected, *ROI* region of interest, *FOG* freezing of gait, *pts* patients, *yr* year(s), *mo* month(s), *d* day(s), *NR* not recorded, *↑* increased, *↓* decreased, − = no correlation, med on = medication on, med off = medication off, stim on = stimulation on, stim off = stimulation off.

### Predictors of motor outcome

The motor symptoms are mainly measured by UPDRS III or MDS-UPDRS III scales. Four studies measured the outcome by percent improvement by comparing UPDRS III total score at presurgical medication-off state (UPDRSIII _med off_) with postsurgical medication-off and stimulation-on state (UPDRSIII _med off, stim on_)^10,19–21^. Among them, one study also measured percent improvement in UPDRSIII at medication-on state (UPDRSIII _med on/ med on, stim on_)^19^. Two studies used the absolute change of UPDRSIII _med off/ med off, stim on_^13,17^. Two studies used the ratio of UPDRSIII _med off/ med off, stim on_^11,14^. Two study using the absolute postsurgical UPDRSIII _med off, stim on_ score^21,22^. One study used percent improvement in postoperative UPDRSIII (UPDRSIII _med off, stim off / med off, stim on_)^23^. One study regressed postoperative scores on variables adjusting for presurgical scores^12^.

#### Cardinal motor symptoms

##### Cortical thickness

**Fig. 3:**
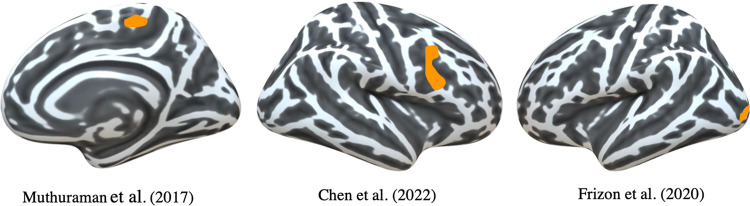
Regions associated with motor outcome in cortical thickness analysis. Reduced cortical thickness in paracentral area and superior frontal region, right precentral cortex and lateral occipital cortex were associated with less motor improvement after DBS. The areas are marked in yellow.

Four studies included in our review analyzed the associations of cortical thickness with motor outcomes (Table 1)^10–13^. All the studies used freesurfer to conduct the analysis and the analysis methods were further summarized in supplementary table 1. Positive correlations between cortical thickness in the motor cortex and motor outcome were found in two studies (Fig. 3). Muthuraman et al. found the cortical thickness of the frontal lobe (paracentral area and superior frontal region) predicted the UPDRSIII _med off, stim on_/ UPDRSIII _med off_ and an improved postoperative outcome at low stimulation voltages. In addition, precuneus, superiortemporal, inferiorparietal, and superiorparietal areas in the left hemisphere also predicted better postoperative outcomes at low stimulation voltages^11^. Chen et al. found a positive association between percent improvement in MDS-UPDRSIII _med-off/med-off, stim-on_ and thickness of the right precentral cortex^10^. Frizon et al. found that cortical thickness of the left lateral occipital region was strongly related to the improvement of postsurgical MDS-UPDRS III _med off/med off, stim on_, as confirmed in both region of interest (ROI) analysis and voxel-wised analysis^12^. Additionally, moderate negative correlations were also observed between the MDS-UPDRS III score and the right lateral occipital area, pars opercularis, posterior cingulate, the superior-temporal area in the right hemisphere as well as the lateral orbitofrontal, transverse temporal, and insula regions in the left hemisphere in ROI analysis, which was not confirmed in voxel-wised analysis^12^.

##### Brain volumetry

Ten studies investigating the relationship between volumetric changes and motor improvement were identified^13,14,17,19–25^. One study utilized 2D measurements including the bicaudate ratio (ratio between the inter-caudate and skull distance)^26^, the Evans index (width of the anterior ventricular horn divided by biparietal cranium diameter)^27^, and the third ventricular width (largest width of the third ventricle at the level of the posterior commissure)^14^ to assess subcortical changes^14^. Other studies used automated or semi-automated volumetry to analyze the alterations of the whole brain or ROIs.

Consistent with the findings of cortical thickness, the volume of the frontoparietal cortex assessed by VBM was found associated with DBS response. Jergas et al. found that volumes of the frontoparietal cortical regions were significantly associated with the percent improvement in UPDRSIII _med off/med off, stim on_^20^. The gray matter loss was identified in clusters in the bilateral medial prefrontal cortex, paracingulate and cingulate gyrus, and a large cluster in the left parietal lobe and the angular gyrus in patients with less improvement in UPDRSIII. In addition, smaller clusters of atrophy were found in the right superior frontal gyrus, reaching the supplementary motor area, as well as in the left middle frontal gyrus and the left precuneus in patients with the less motor improvement^20^.

Morphometry of thalamus was also found associated with motor outcomes. Yim et al. showed that patients with larger improvement had increased volumes of the right thalamus, anterior cingulate and the left anterior middle frontal cortex^23^. The results of the thalamus were partially replicated by Younce et al. who found that smaller thalamic volumes and larger ventricular (the lateral and 3rd ventricles) volumes (which were significantly inter-correlated) predicted less absolute improvement in UPDRS III _med off/med off, stim on_ after STN-DBS^13^.

While Younce et al. demonstrated the predictive capability of the ventricle volume (containing the lateral and 3rd ventricles) in motor outcome after bilateral STN-DBS^13^, Price et al. evaluating patients receiving unilateral STN-/GPi-DBS showed that ventricle volume (as measured by the lateral ventricle) and the measurements of contralateral/ipsilateral volumes to the side of symptom onset or DBS lead placement did not predict absolute improvement in UPDRSIII _med off/med off, stim on_ or axial motor changes measured by UPDRS subscores^17^. Also, patients with higher motor improvements did not have smaller ventricles relative to those with less motor improvement^17^. Another study used the bicaudate ratio, the Evans index, and the third ventricular width to measure subcortical volumes. The indices were able to predict postsurgical UPDRSIII_med off, stim on_ /UPDRSIII _med off_ for patients receiving bilateral pallidal DBS^14^. The Evans index and third ventricular width also correlated with presurgical medication response.

#### Axial symptoms

Two studies focused on the axial symptoms of PD patients^24,25^. Karachi et al. found that PD patients with developed or aggravated freezing of gait (FOG) within 1 year (as measured by the subitem of UPDRS II) showed significantly reduced bilateral gray matter density in the sensorimotor and associative putamen, and an increased gray matter density in the left inferior frontal and right inferior temporal gyri^24^. PD patients with falls after surgery had significantly reduced gray matter density in the left postcentral gyrus and an increase in the culmen of the right cerebellar lobe relative to non-falling PD patients. Statistical maps of stimulation showed that the best effects of STN-DBS on FOG and falls were associated with the location of contacts within the STN, but no specific locations were related to aggravation^24^. Additionally, the atrophy of basalis of Meynert (NBM) showed a trend for predicting the degree of increase in swing time variability after 3 years of continuous STN-DBS^25^.

### Predictors of non-motor outcome

13 studies included in our review evaluated the relationship between brain morphometry and non-motor symptoms after DBS, with nine studies investigating cognitive abilities and five studies focusing on psychiatric complications (Table 2).

**Table 2:** Studies investigating the associations between structural MRI features and non-motor outcomes in our review.

Author; year	Pts (male/female)	Duration of disease, yr	Age, yr	Associations	LFU	UPDRS III before	UPDRS III after
Cognitive decline							
Aybek; 2009^28^	14 (/70) PD with PDpD (9/5) with paired control group STN	PDpD:15.0 ± 5.1 PDnpD:14.4 ± 4.4	PDpD:69.2 ± 5.7; PDnpD:66.6 ± 6.6	↓ Preoperative hippocampal volumes in PDpD	≥2 yr, 40 ± 16 mo	Med off: PDpD:44.4 ± 11.8 PDnpD:39.7 ± 10.5 Med on: PDpD:21.7 ± 6.9 PDnpD:22.7 ± 9.1	Med off: PDpD:27.9 ± 12.4 PDnpD:23.3 ± 8.8
Geevarghese; 2016^30^	40 (23/17) BL STN	11.9 ± 5.0	60.2 ± 7.2	↑ Volumes of the left and right hippocampus and left thalamus & Improvement in List Learning score. ↓ Volumes of the left and right thalamus and left and right hippocampus in pts in the decline group for the Delayed Story Recall test relative to those in the stable group.	8.8 ± 2.0 mo	Med off: 39.2 ± 19.1	NR
Blume; 2017^29^	40 (30/10) BL STN	12.5 ± 4.5	61.8 ± 6.7	↑ WML & Rapid onset of dementia within 1 yr. ↑ WML & Increased rate of decline in cognitive composite score within 3 yr.	3 yr	Med off: 33.6 ± 10.7; Med on:12.2 ± 6.6	Med NR: PDpD after 3 yr:34.8 ± 11.3 NC or MCI after 3 yr:30.2 ± 7.8
Puy; 2018^31^	Case study (1, female) STN	7	68	A medial temporal lobe atrophy score of 2 of the patient who developed dementia after deep brain stimulation.	/	Med off:30	/
Planche; 2018^15^	42 (26/16) BL STN	10 [6–17]	64 [45–70]	↓ The left nucleus accumbens & The variation of the initiation/perseveration subscore of the MDRS. ↑ The left lateral ventricle & The variation of the total MDRS score/ the initiation/perseveration subscore. ↓ The right and left superior frontal gyrus thickness & The variation of the backward digit-span task.	1 yr	Med off: 32 [8–70]; Med on: 10 [3–28]	Med off: 21 [7–53]; Med on: 13 [2–41]
Weinkle; 2018^32^	43 (30/13) 29 BL STN 14 UL STN	9.4 ± 4.1	62.3 ± 7.3	↑ WML & Declined performance on the Block design visuospatial task. ↑ Volume of the right hippocampus volume & Improvement in CVLT-II recognition hits score.	14.4 ± 6.4 mo	NR	Percent change in UPDRS score: −36.8 (± 27.4)
Lu; 2021^22^	59 (33/26) 57 BL STN 2 BL GPi	8.2 ± 4.3	PD:65.7 ± 7.5; Control: 59.8 ± 7.1	↑ The left STN volume & Higher preoperative MMSE and MoCA scores.	6 mo	NR	NR
Kübler; 2022^33^	55 (39/16) BL STN	10.8 ± 4.7	61.4 ± 7.5	↑ NBM volume & Improved cognitive outcome measured by MMSE or DemTect score.	1 yr	Med off: 44.2 ± 14.4	Med off: 25.0 ± 12.7
Psychiatric changes							
Bourne; 2012^16^	Confusion 21(/226); 21 controls 14 BL/UL STN 2 BL/UL GPi 5 BL/UL VIM	NR	Confusion:65.7; Control :65.6	↑ Minimum width of the lateral ventricles in pts with postoperative confusion relative to pts without.	/	NR	NR
Hrabovsky; 2017^18^	80 (50/30) BL STN	Pts without mental alterations: 11.0 [7.0, 15.0]; Pts with mental alterations: 10.5[7.0,17.9]	61.8 [50.0, 69.8]	The third ventricular length, or inter-mammillary distance did not correlate with early postoperative mental status alterations.	>7 d	NR	NR
Tanaka; 2018^34^	61 (27/34) 52 STN; 8 GPi; 1 VIM; (4 UL; 57 BL)	NR	65.6 ± 9.2	↓ Volumes of the total WM and WM in the temporal stem & Increased duration of POD.	/	NR	NR
Wang;2019^35^	Nondelirium :133(65/68); Delirium:32(21/11) BL STN	Nondelirium: 9.4 ± 4.5; Delirium: 9.8 ± 4.0	Nondelirium: 60.1 ± 9.1; Delirium: 62.8 ± 9.3	Preoperative brain atrophy & occurrence of POD after DBS.	/	Nondelirium: 50.2 ± 13.6; Delirium: 55.9 ± 14.6	NR
Radziunas; 2020^36^	22 (10/12) BL STN; 18 healthy controls	Pts without neuropsychiatric complications: 10.4 ± 4.0; Pts with neuropsychiatric complications: 13.5 ± 2.5	58.0 ± 8.2	↓ White matter volume, cortical thickness and cortical area of the left caudal middle frontal area & Increased incidence of neuropsychiatric complications.	/	Med on: 17.4 ± 6.1	NR
Lu; 2021^22^	59 (33/26) 57 BL STN 2 BL GPi	8.2 ± 4.3	PD:65.7 ± 7.5; Control: 59.8 ± 7.1	↓ The left and right STN volume & Increased postoperative HAMD score.	6 mo	NR	NR

*PDpD* PD patients with postsurgical dementia, *PDnpD* PD patients without postsurgical dementia, *POD* = postoperative delirium, *STN* subthalamic nucleus, *GPi* internal globus pallidus, *VIN* ventral intermediate nucleus, *NBM* nucleus of basalis of Meynert, *BL* bilateral, *UL* unilateral, *WML* white matter lesions, *ICV* intracranial volume, *UPDRS* Unified Parkinson’s Disease Rating Scale, *CVLT-II* The California verbal learning test-II, *HAMD* Hamilton Depression Scale, *MMSE* Mini Mental Status Examination, *MDRS* Mattis Dementia Rating Scale, *MOCA* Montreal Cognitive Assessment, *pts* patients, *yr* year(s), *mo* month(s), *d* day(s), *NR* not recorded, *↑* increased, *↓* decreased; − = no correlation, med on = medication on, med off = medication off, stim on = stimulation on, stim off = stimulation off.

#### Cognitive decline

Different outcome measures of cognitive decline including clinical scales (Mini Mental Status Examination, MMSE; Mattis Dementia Rating Scale, MDRS), indexes of specific cognitive domains as well as diagnostics criteria (the DSM-IV dementia criteria, the Movement Disorders Society Task Force Criteria level 2)^28,29^ were adopted in studies evaluating cognitive functions. The follow-up duration varied from 6 months to over 2 years. Different covariates were considered in the studies and were collected in Supplementary Table 2.

##### Brain volumetry

Eight studies that were included in our review reported associations of brain volumetry with the cognitive outcome^15,22,28–33^. ROI analysis assessing specific structures was adopted in these studies. (Fig. 2).

A case report from Puy et al. highlighted the association of hippocampus volume with dementia after DBS^31^. Replicated results were found in the prediction of cognitive decline by reduced hippocampal volumes^28,30^. Compared with the age and gender-matched control group who underwent STN-DBS but did not develop dementia after surgery, patients who developed dementia had significantly smaller presurgical hippocampal volume. Logistic regressions showed that hippocampal volume was an independent predictor of dementia. Hippocampal volumes were significantly associated with postoperative MMSE in the whole population but were not related to memory scores or executive function scores^28^.

In the evaluation of specific verbal memory functions of PD patients, Geevarghese et al. found that hippocampal (left and right) and thalamic volumes (left) were significant predictors of changes in List Learning score after STN-DBS. Additionally, patients with a more severe decline in the Delayed Story Recall test had significantly smaller thalamic and hippocampal volumes compared to those in the stable group^30^.

Apart from the hippocampus and thalamus, atrophy of the nucleus accumbens was also suggested to correlate with cognitive decline after DBS^15^. The atrophy of left nucleus accumbens predicted postoperative decline in executive functions of patients after STN-DBS in 1 year^15^. Presurgical left nucleus accumbens volume was strongly correlated with the variation in the MDRS initiation/perseveration subscore. The volume of the presurgical left nucleus accumbens also correlated with the right and left ventricle volumes. Additionally, correlations were found between the variation of total MDRS and the left lateral ventricle volume, between the variation of the initiation/perseveration subscore and the left lateral ventricle volume, between the variation of the backward digit-span task and the right and left superior frontal gyrus thickness, after adjustments for age, gender, disease severity, decrease in L-DOPA equivalent dose following surgery and “anterior” contact location. No significant association between contact location and cognitive decline was found in the study.

The volume of NBM was correlated with the relative change of MMSE or DemTect scores of PD patients 1 year after STN-DBS^33^. The results were demonstrated in both the regression model and the machine learning model, where NBM volume was the most predictive variable. The cognitive outcome was significantly influenced by the NBM/total intracranial volume ratio, but not by the number of cognitive domains affected presurgically nor UPDRS III _med off_. Nevertheless, only a small amount of variability could be predicted by NBM size (R^2^ of 0.149 in a multiple linear regression model), which needs further validation from larger cohorts.

#### Psychiatric changes

Of the six studies that evaluated psychiatric changes, five studies focused on immediate psychiatric alterations^16,18,34–36^, and only one study evaluated post-operative clinical scales including neuropsychiatric assessments after 6 months^22^. Most studies evaluated postoperative confusion, delirium, or impulsivity, whereas long-term mood disturbances such as apathy and depression lacked attention.

Brain atrophy was recognized as an independent risk factor for post-operative psychiatric changes^16,35,36^. Patients who developed neuropsychiatric complications (7/22) had significantly smaller gray matter thickness and white matter volume when compared to patients without acute postoperative neuropsychiatric complications^36^. Subsequent analysis showed that, patients with acute neuropsychiatric changes had significantly smaller white matter volumes in the left caudal middle frontal gyrus, left lingual gyrus, left pericalcarine gyrus, and left precuneus, as well as significantly reduced cortical thickness bilaterally in the frontal lobe (caudal middle frontal and precentral gyruses), temporal lobe (inferior and middle temporal gyruses) and parietal lobe (postcentral, superior parietal and supramarginal gyruses). Significant cortical atrophy was found on the left-brain hemisphere in caudal middle frontal and inferior temporal areas^36^. Wang et al. found that presurgical brain atrophy as visually inspected was an independent factor that influenced the occurrence of postoperative delirium (POD)^35^. Bourne et al. showed that patients with post-operative confusion after DBS for the treatment of various diseases including PD had a significantly larger minimum width of the lateral ventricles than that of the controls^16^. Additionally, greater maximum and minimum width of the lateral ventricles were also associated with a higher occurrence of other complications. However, the correlation demonstrated between changes in ventricle morphometry and psychiatric complications was not consistent^18^. Increased inter-mammillary distance (>8 mm) was found in five PD patients older than 60 years old, among whom the incidence of early postoperative mental changes reached 60%^18^.

At the basal ganglia level, Lu et al. found that the left STN volume differed significantly between PD patients and healthy control subjects and was negatively correlated with postsurgical Hamilton Anxiety Scale and Hamilton Depression Scale (HAMD) score. The right STN volume was negatively correlated with the post-operative HAMD score. The lateralized difference of STN may be explained by the fact that 46/59 patients in the study had disease onset in the right limb, and most patients exhibited more severe right limb symptoms^22^.

## Discussion

This systematic review investigated the associations between brain morphometric features and clinical outcomes of PD patients following DBS treatment: A total of 25 articles with 1316 patients containing structural MRI data were identified and reviewed. Replicated results showed that reduced brain volume/cortical thickness in the motor cortex and the thalamus were associated with less improvement in cardinal motor symptoms, while other structures including anterior cingulate and brain ventricles were also suggested to hold contributory roles. Regarding non-motor outcomes, reduced volume of the hippocampus was repeatedly reported to associate with poor cognitive performance after DBS, while other structures including the nucleus accumbens, thalamus, and ventricles were also reported. NBM was suggested to predict cognitive performance and axial symptoms after DBS surgery. Different anatomical features are responsible for specific or joint pathophysiological functions related to clinical manifestations.

The findings of brain morphometry related to DBS response of cardinal motor symptoms mainly resided in sensorimotor cortex, thalamus and brain ventricles. The sensorimotor cortex constitutes a critical component of the hyperdirect pathway. Antidromic modulations from targets of DBS of the pathway were evidenced by both human and animal studies^37^. Connectivity analysis showed that structural and functional connectivity to this region was associated with the clinical improvement of DBS^38–42^. The reduced cortical thickness and diminished volume of this region were also reported to correlate with motor outcomes in our review^10,11^. However, the finding was not replicated except for two analyses which found a non-significant correlation between the volume of sensorimotor regions and motor response^13,23^. The sensitivity of the method may be a reason. Regional ROI analysis can fail to detect changes in small clusters as determined by voxel-wised analysis. The study conducted by Chen et al.^10^ used the same clinical scale (MDS-UPDRS) and had a similar disease duration with that of Frizon’s study^12^, while the number of subjects enrolled was twice as many^12^ (73 vs 36), of which the analysis should therefore be more sensitive.

Ventroanterior and ventrolateral motor thalamic areas are critically positioned in the pallidal and cerebellar thalamocortical pathways^43,44^. The reduced thalamic volume was associated with poor DBS outcome, which was alerted in three of our included studies^13,23,30^. Thalamus comprises multiple subnuclei with different connections and functions^45^. Morphological changes in thalamic subnuclei were associated with clinical symptoms of PD individuals and have been proposed to predict clinical improvement^46^. Nevertheless, none of the existing studies have investigated the associations of thalamic subregions with DBS effects. Structural assessments of thalamus subnuclei can be achieved by conventional structural MRI, the value of which is yet to be explored.

Ventricle volume is regarded as a good indicator of brain atrophy^13,14^. It allows the estimation of disease progression and is correlated with atrophy of other critical brain areas^47,48^. Additionally, the enlargement of ventricles was associated with operative problems such as cerebrospinal fluid leakage^49–51^ and was considered the most reliable predictor for brain shift^52^. Four studies included in our review supported the role of ventricle volume in the prediction of DBS outcomes^13–16^ whereas two studies did not support its value^17,18^. Several differences should be noted in the studies. First, the calculation of ventricle volumes was handled by different methods (linear measurements or automated segmentation algorithms). Second, the definition of brain ventricles differed. Younce et al. evaluated ventricle volumes containing the lateral and third ventricles^13^ whereas Price et al. investigated only the lateral ventricles^17^, which yielded contradictory results. Third, different study designs may hamper cross-comparisons. Differences in Younce et al.’s and Price et al.’s studies were also observed in surgery selections (bilateral STN DBS vs unilateral GPi/STN DBS), patient cohorts (*n* = 86, age mean = 62.9, UPDRSIII 36 ± 10 vs *n* = 37, age mean = 58.8, UPDRSIII 44 ± 11) and follow-up durations (15 vs 4 months), which should all be noted while interpreting the results.

Aside from the above regions, other indicators that are suggested to predict cardinal motor improvement of DBS include (1) Lateral-occipital cortex, which contains the extrastriate body area responsible for the visual identification of body parts and movements^12^. Functional imaging analysis showed that the area may play a compensatory role in PD for impaired motor functions performed by the premotor cortex^53^, while reduced cortical thickness may disrupt the compensatory activity. (2) Anterior cingulate gyrus, an important cortical component of the motor circuit responsible for various movement disorders^23,54^. Probabilistic tractography studies showed that the cingulate gyrus was composed of the anatomical network with the frontal and prefrontal cortex, whose dysfunction may alter the connectivity between the STN and frontal lobe^55^.

Axial symptoms are largely resistant to dopaminergic therapy and respond variably to DBS treatment. Some symptoms such as gait impairment and FOG seem to be partially improved by DBS, whereas others such as postural instability and related falls might worsen after surgery^56^. Growing evidence suggests the degeneration of cholinergic neurons, a pathological hallmark of progressive cognitive deterioration, is crucial in the pathophysiology of gait and balance disorders^57^. Studies involved in our systematic review suggested the role of NBM^25^ and several other cortical and subcortical regions^24^ in associations with gait impairment and falls after DBS surgery. Patients with NBM degeneration showed a significant cognitive decline, which was also associated with higher axial scores, highlighting the relationship between axial symptoms and cognitive functions^58^. The findings shed light on the understanding of gait and balance control in PD patients.

Although DBS does not seem to affect the overall cognitive profile in PD patients, it is associated with focal cognitive deficits^59^. Apart from the limbic and cortical spread of Lewy body pathology, several factors contribute to the cognitive deterioration of PD patients including neuronal loss, neurotransmitter deficits, and cerebral network disruptions^60^. These can be partially reflected by imaging parameters, which provides potential biomarkers for the prediction of non-motor symptoms in supplement to neuropsychological evaluations. Hippocampus is a major structure responsible for complex episodic memory processes, the atrophy of which is an established biomarker of degenerative processes such as AD^61^. The involvement of HA in the progression of Lewy bodies contributes significantly to the development of dementia in PD, which is demonstrated in imaging, proteomics and biochemical analysis^62–64^. Four studies identified in our review noted the associations of reduced presurgical hippocampal volume with verbal memory decline or overall cognitive impairment after DBS, suggesting hippocampal atrophy as a preexisting risk factor for cognitive decline for patients receiving DBS surgery^28,30–32^. Connections between hippocampal subfields with cognitive functions and motor subtypes of PD patients have been proposed, the study of which might exert increased potential in patient selection with candidate stratified to different motor subtypes^65,66^. Other brain structures were also reported to correlate with the cognitive outcome of DBS, including thalamus^30^, nucleus accumbens^15^ and NBM^33^. These structures provide important crossroads for cognition-relevant information processing. As the progress of cognitive deterioration is increasingly impacted by cholinergic pathology, shrinkage of the central node in the cortical cholinergic system may disturb cognitive functions, which may be due to the imbalance of neurotransmitter systems^67,68^.

Treatment effects of DBS are curtailed by psychiatric side effects, which include transient confusion in the brief postoperative period, and depression, apathy, and hypomania that may occur in a longer follow up^69^. Psychiatric disorders are frequently observed in post-DBS treatment where post-operative delirium (POD) in the immediate duration after STN-DBS surgery is seen in 22% of patients^70^ and symptoms such as depression and anxiety are observed in up to 50% of patients^71^. Most of the studies included in our review focused on brief mental alterations. These studies found that brain atrophy, as visually inspected^35^ or measured by the width of lateral ventricles^16^, white matter volume^34^ and frontal cortical regions^36^ were associated with immediate psychiatric symptoms. Caudal middle frontal area was suggested to correlate with POD after DBS^36^. The area has been linked with impulse control disorders in PD and brief psychotic disorder^72,73^. Atrophy of the region may impair both short intralobar connections and top-down inputs to other brain structures, which is considered important pathophysiology of major psychiatric disorders^74,75^. STN was found to associate with depression after 6 months of DBS^22^. The anterior limbic part of STN has numerous connections with limbic circuits according to the classic tripartite STN organization model, although recent ultrahigh-resolution MRI studies suggest cortical inputs of STN are overlapping^76,77^. Mechanical STN lesioning can cause limbic circuitry imbalance exhibited by depression-like behavior in rats^78,79^. The atrophy of STN may indicate the impairment of the limbic circuitry, while its predictive function remains to be determined.

Several limitations apply to this study. First, 7/25 studies included in this review did not consider the influence of confounding variables (age, disease duration, cognitive status, presurgical levodopa usage, contact locations, DBS procedure and so on) apart from intracranial volume. Especially, only five studies targeting STN considered post-operative lead position in their analysis. The results suggested that the contact locations and the consequent overlap between the motor STN and the individual patient’s VTA were associated with the outcome. It is still worthy of disentangling the variances, either with a multivariate model or building a matched control group, in outcome explained by presurgical morphometry from the ones explained by postsurgical lead locations in future cohorts. Second, only one study with 34 patients in our review analyzed GPi-DBS related brain morphometry separately from STN-DBS^14^. So far, the morphometric findings are largely inconsistent between studies on different targets or even between those on a single target, which is more likely to be attributed to the limited sample size and varied analytic methodologies than the target itself. In the future, the findings are yet to be validated with a larger sample size of patients with both GPi- and STN- DBS. Third, as described previously, the definition of motor improvement varied among studies. Comparisons of motor scales before and after surgery may reflect the overall benefit of DBS surgery while comparing postsurgical motor scales between stimulation-on and stimulation-off states can minimize the microlesion effect and better represent the short-term stimulation effect.

This study summarizes the state-of-the-art evidences in the association between structural MRI based on T1-weighted sequence and clinical outcome of DBS in PD. Reduced cortical thickness/brain volume of motor cortex and thalamus were associated with less motor improvement. Reduced volume of the hippocampus was associated with impaired cognitive performance after DBS. Other indicators such as the cortical thickness of the lateral-occipital cortex, volumes of the anterior cingulate gyrus, brain ventricles, nucleus accumbens, STN and NBM have also shown predictive utility for postsurgical outcomes. The findings of this systematic review facilitate the construction of applicable prognostic models to assist in the major issues of patient selection and promote the understanding of the DBS mechanism. Due to the relatively small number of subjects and generally inconsistent results, the findings are yet to be validated with a larger sample size and appropriate analytic procedure.

## Methods

### Search strategy

This review was conducted following the Preferred Reporting Items for Systematic Reviews and Meta-Analyses (PRISMA) statement. We searched the literature in the online databases of PubMed and Embase from inception to April 5, 2022. The MESH terms (PubMed), EMTREE terms (Embase), and keywords we used were as follows: Parkinson’s disease AND stimulation AND (gray matter OR thickness OR volume OR atrophy OR morphometry OR morphology OR structural covariance). Additionally, we scanned the reference lists of included studies and relevant reviews for additional relevant cited articles.

### Eligibility criteria

The inclusion criteria were: (1) written in English; (2) full-text articles; (3) included participants with PD; (4) examined the outcome of DBS; (5) contained presurgical morphometric features; (6) examined the relationship between the outcome of DBS and presurgical morphometric features. Studies were excluded if they (1) were conference abstracts, correspondence, editorials, meta-analysis, systematic reviews, reviews, or chapters; (2) were not in English; (3) were animal or other in vitro studies; (4) were simulation studies; (5) did not assess the outcome of deep brain stimulation; (6) did not assess presurgical brain morphometric features; (7) were not relevant as considered by the authors. DBS procedures were summarized in supplementary table 3.

### Selection and data extraction process

The following information was extracted: year of publication, sample size, study type, participant demographics including age and sex, disease duration, pre- and post-surgical Unified Parkinson’s Disease Rating Scale (UPDRS) III or Movement Disorder Society (MDS)-UPDRS III scores, morphology indices (brain volume or cortical thickness) and covariates used in the analysis. Two authors (Wang and Lai) screened the abstracts and two authors (Wang and Li) collected the data independently.

## Supplementary information

Supplementary information

## Data Availability

No datasets were generated or analyzed during the current study.

## Code availability

No codes were generated or analyzed during the current study.
