# Supplementary material for: A systematic review of brain morphometry related to deep brain stimulation outcome in Parkinson’s disease
Source: NPJ Parkinsons Dis. 2022 Oct 13;8:130. doi: 10.1038/s41531-022-00403-x (PMC9556527; doi:10.1038/s41531-022-00403-x)
Supplement: Supplementary file 1 — Supplementary information [file 41531_2022_403_MOESM1_ESM.pdf]

Supplementary Table 1 Studies investigating the associations between structural MRI features and motor outcomes

topic	Author; year	Main findings	Imaging analysis methods	Sequence	Magnetic field strength	Covariates	Outcome measurements
Motor outcome; quality of life	Bonneville; 2005	1).The mesencephalon surface predicted the postoperative residual ADL score $_{\text{med off/on, stim on}}$. 2).Volume of the caudate nucleus predicted the pre- and postoperative LED.	ROI by manual/automated segmentation followed by automated or manual measurement.	T1:TR/TE/TI: 15/5/600 ms, FoV: 240 mm, matrix: 256×256, 1.5 mm thick, acquisition time: 10 min T2:TR/TE: 2200/90 ms ,FoV: 28, matrix: 256×192, acquisition time: 11 min	1.5T	NR	improvement rate of UPDRSIII$_{\text{med off/med off, stim on}}$； postoperative UPDRSIII $_{\text{med on/off, stim on}}$
Motor outcome; quality of life	Hamasaki; 2010	1).The WMF positively correlated with percent improvement in the total UPDRS, UPDRS II and UPDRS part III $_{\text{med off/med off, stim on}}$. 2).The WMF positively correlated with postoperative total UPDRS, UPDRS II, UPDRS III $_{\text{med on, stim on}}$.	brain volume using VBM by SPM5	TR/TE/TI: 1,900/4.7/900 ms; imaging time, 4 min and 18 s. FoV: 23×23 cm, matrix:256×256, one excitation	3T	NR	improvement rate of postsurgical UPDRSIII scores $_{\text{med off/on, stim on}}$ compared to presurgical UPDRSIII $_{\text{med off/on}}$
Motor outcome; axial symptoms	Price; 2011	Lateral ventricular volume did not correlate with absolute improvement of UPDRS III $_{\text{med off/med off, stim on}}$ or axial symptoms.	lateral ventricular volumes by semi-automated segmentation; Intracranial volume using Brainsuite	typical TR/TE/TI: 11/3.87/600, FA:15°, 1.5mm thick, 120 slices	1.5T	Age, education, duration of PD symptoms, pre-surgery disease severity, and baseline general cognitive status	residual change score of UPDRSIII $_{\text{med off/med off, stim on}}$

Motor outcome	Muthuraman; 2017	1). Increased cortical thickness of paracentral area and superior frontal region predicted the improvement of UPDRSIII $_{\text{med off/med off, stim on}}$. 2). In pts with reduced cortical thickness of these areas, a higher voltage was needed for an optimal clinical response.	cortical thickness using vertex-by-vertex and ROI analysis by freesurfer	TR/TE:7.7/3.6 ms, FA: 8°, 160 slices, 1mm thick, matrix: 256× 256mm, isotropic resolution:1×1×1mm	3T	age and disease duration	postoperative UPDRSIII $_{\text{med off, stim on}}$ / preoperative UPDRSIII $_{\text{med off}}$
Motor outcome	Younce; 2019	Decreased ventricular volumes and increased thalamic volumes predicted the absolute improvement of UPDRS III $_{\text{med off/med off, stim on}}$.	brain volumes and cortical thickness using ROI by freesurfer	TR/TE/TI: 2400/3.14/1000 ms, FA:8°, 0.9 mm^3 voxels	3T	age, disease duration, level of education, LED, and baseline UPDRS score	changed score of UPDRSIII $_{\text{med off/med off, stim on}}$
Motor outcome; quality of life	Frizon; 2020	1).Cortical thickness of the left lateral-occipital cortex correlated with the improvement of MDS-UPDRSIII $_{\text{med off/med off, stim on}}$ in ROI analysis and vertex-wise analysis. 2).Greater cortical thickness in frontal and temporal regions may correlate with increased postoperative improvements in UPDRS II (not confirmed by vertex-wise analysis)	cortical thickness using ROI and vertex-wise analysis by freesurfer	TR/TE:11/4.6 ms, FA:25°, 192 slices,matrix size:256 × 256 mm, voxel size:1 × 1 × 1 mm^3	3T	Age, handedness, dementia rating score and disease duration as candidte covariates.	ROI analysis: Regress the MDS-UPDRSIII score $_{\text{med off, stim on}}$ while adjusting for preoperative UPDRSIII $_{\text{med off}}$
Motor outcome; levodopa response	Hamed; 2020	Increased bicaudate ratio, the Evans index, and the third ventricular width correlated with increased postsurgical UPDRSIII$_{\text{med off, stim on}}$ /preoperative UPDRSIII $_{\text{med off}}$.	bicaudate ratio, Evans index, and third ventricular width	NR	1.5T	NR	postoperative UPDRSIII $_{\text{med off, stim on}}$ / preoperative UPDRSIII $_{\text{med off}}$

Motor outcome	Yim; 2020	1). Increased volumes of the anterior cingulate and right thalamus volumes (after FDR correction) and increased volumes of right nucleus accumbens, lef anterior middle frontal gyrus (not after FDR correction) were observed in the higher MI group.<br>2). Increased volumes of the left caudate, paracentral, right primary sensory and left primary motor cortex (not after FDR correction) were observed in the lower MI group.	brain volume using ROI by NeuroQuant and VBM by SPM12	TR/TE:7.7/3.6 ms; FA: 8°; 160 slices,1 mm thick; matrix:256×256 mm; isotropic resolution:1×1×1 mm	1.5T	age, sex, and ICV	(Postoperative UPDRS $_{\text{med off, stim off}}$−Postoperative UPDRS $_{\text{med off, stim on}}$]/Postoperative UPDRS $_{\text{med off, stim off}}$)*100
quality of life; motor outcome; depression; cognitive decline	Lu; 2021	1).The left STN volume negatively correlated with pre- and postoperative quality of life scores, post-operative HAMD score, and positively correlated with preoperative MMSE and MoCA scores.<br>2).The right STN volume negatively correlated with the post-operative HAMD score.<br>3).The left or right STN volume, or ICV did not correlate with pre- and post- UPDRSIII scores.	regional brain volume using ROI by freesurfer v5.1.0	TR/TE:7.0/3.0 ms, FA : 12°, FoV:256 × 256 mm^2, matrix size:256 × 256, 1mm thick, and number of slices:192; the scanning time was 4 min 15 s	3T	ICV	postoperative UPDRSIII score $_{\text{med on/off, stim on/off}}$
Motor outcome	Chen; 2022	1).Increased cortical thickness of the right precentral cortex correlated with percent improvement in MDS-UPDRSIII $_{\text{med off/med off, stim on}}$.	cortical thickness using vertex-wise analysis by freesurfer;brain volume using VBM by freesurfer	TR/TE: 6.6/3.1 ms, FA: 8°, matrix size: 240 × 240, isotropic voxel:<br>1 × 1 × 1 mm^3, number of slices:	3T	age, sex, and VTA	improvement rate of MDS-UPDRSIII $_{\text{med off/med off, stim on}}$

		2). No subcortical gray or white matter volumes correlated with the percent improvement in MDS-UPDRSIII $_{med\ off/med\ off,\ stim\ on}$.		196			
Motor outcome; levodopa response	Jergas; 2022	1).The morphometric estimates did not correlate with the response to levodopa.<br>2).Increased volume of the frontoparietal cortex correlated with the percent improvement in UPDRSIII $_{med\ off/med\ off,\ stim\ on}$.	Brain volume using VBM by Statistical Parametric Mapping Toolbox V12 and CAT12	Given in detail at the individual level	3T	age, sex, ICV, disease duration, scanner type, mSTN-overlap,TR and TE	improvement rate of UPDRSIII $_{med\ off/med\ off,\ stim\ on}$
Fall and FOG	Karachi; 2019	1).Relative to other pts groups, pts with FOG worsening had putamen grey matter density decrease, and fallers pts a left postcentral gyrus atrophy.<br>3).Effects of STN-DBS on FOG and falls correlated with the location of contacts within the STN. No specific location related to aggravation.	brain volume using VBM by SPM8	NR	NR	Age for all analyses and MDRS cognitive score only for falls	\
Gait variability	Wilkins; 2020	1).Decreased NBM volume correlated with larger swing time variability and increased stride time.<br>2).STN-DBS and dopaminergic medication improved cardinal motor signs and gait speed but not swing time variability.<br>3).NBM atrophy showed a trend for predicting the degree of increase in swing time variability.	brain volume using VBM by SPM12	TR/TE: 8.24/3.24 ms, FoV:240x240mm^2	3T	age, sex, and disease duration	\

STN= subthalamic nucleus; NBM = nucleus of basalis of Meynert; WMF = white matter fraction; GMF = gray matter fraction; CSFF = cerebrospinal fluid fraction; ICV = intracranial volume; UPDRS = Unified Parkinson's Disease Rating Scale; HAMD = Hamilton Depression Scale; MMSE = Mini Mental Status Examination; MDRS = Mattis Dementia Rating Scale; MOCA = Montreal Cognitive Assessment; PDQ-39 = 39-item Parkinson’s Disease Questionnaire scale; LED = L-DOPA equivalent dose; FDR = false discovery rate; ROI = region of interest; FOG = freezing of gait; TR = repetition time; TE = echo time; TI = inversion time; FoV = field of view; FA = flip angle; BW =bandwidth; pts = patients; yr = year ; mo = month; d = day; min = minute; ms = millisecond; NR = not recorded; med on = medication on;med off = medication off; stim on = stimulation on; stim off = stimulation off.

Supplementary Table 2 Studies investigating the associations between structural MRI features and non-motor outcomes

topic	Author; year	Main findings	Imaging analysis methods	Magnetic field strength	Sequence	Covariates
Cognitive decline	Aybek; 2009	1).PDD had smaller preoperative HV than PDnD. 2).Every 0.1 ml decrease of HV increased the likelihood to develop dementia by 24.6%.	hippocampal volume using mannual segmentation and total brain volume using automatic segmentation	1.5T	NR	MMSE scores, the severity of the disease in populations matched for age and gender
Cognitive decline	Geevarghese; 201640	1).Increased volumes of the left and right hippocampus and the left thalamus correlated with changes in List Learning score. 2).Decreased left and right thalamic and left and right hippocampal volumes were observed in pts in the decline group for the Delayed Story Recall test relative to those in the stable group.	brain volume using FSL	1.5T	TR/TE/TI:8.4/3.5/450 ms, FA:25°, BW: +/- 23 KHz;FoV: 300 mm, 1 excitation, matrix:256 x 256, and 1.5 mm thick	age, duration of symptoms and disease severity as potential confounders
Cognitive decline	Blume; 2017	1).Pts with a rapid onset of dementia within one, respective three yrs following DBS showed significant higher WML volumes compared to cognitive normal and MCI pts. 2).WML volume correlated with the rate of decline in cognitive composite score within three years after DBS surgery.	WML using iPLAN software	1.5T	SL:30.50;ST:5.00; resolution: 256×192; TR/TE: 7530.00/110.00 ms; FoV:230.00×172.50 mm	age, gender and PD disease duration
Cognitive decline	Puy; 2018	Reexamination of the presurgery brain MRI gave a medial temporal lobe atrophy score of 2 of the patient who	medial temporal lobe atrophy (Schelten) score	/	/	/

		developed dementia after DBS.				
Cognitive decline	Planche; 2018	Correlations were found:<br>(1) between the variation of the total MDRS score and left lateral ventricle volume;<br>(2) between the variation of the initiation/perseveration subscore of the MDRS and the left nucleus accumbens volume and the left lateral ventricle volume;<br>(3) between the variation of the backward digit-span task and the right and left superior frontal gyrus thickness.	subcortical and hippocampal volume using ROI by Volbrain; cortical thickness using ROI by freesurfer	1.5T	TR/TE/TI :1870/2.97/1100 ms, resolution:0.625×0.625×1.3 mm, FoV:256×228 mm .	age, gender, pre-operative disease severity, change in dopaminomimetic dose after surgery and contact location
Cognitive decline	Weinkle; 2018	1).WML volume correlated with the decline in performance on the Block design visuospatial task.<br>2).Right hippocampus volume positively correlated with the change in performance on the CVLT-II recognition hits score.<br>3).WML volume did not correlate with other changes in performance on cognitive measures in the domains of memory, verbal fluency, executive function, or attention.<br>4).Presurgical forebrain parenchyma and hippocampal volumes did not correlate with other declines in performance on cognitive measures.	WML using LST toolbox; Brain volumetry using ROI by Neuroquant	NR	NR	age at baseline, education at baseline, number of vascular risk factors, disease duration, percent change in UPDRS, postsurgical LED, and time from surgery to postsurgical NP testing.

Depressio;Cognitive decline	Lu; 2021	1).The left STN volume was smaller in PD pts compared to healthy controls.<br>2).The left STN volume negatively correlated with pre- and postoperative quality of life scores, post-operative HAMD score, and positively correlated with preoperative MMSE and MoCA scores.<br>3).The right STN volume negatively correlated with the post-operative HAMD score.<br>4).ICV positively correlated with preoperative MMSE score.<br>5).the left or right STN volume, or ICV did not correlate with pre- and post- UPDRSIII scores.	regional brain volume using ROI by freesurfer	3T	TR/TE:7.0/3.0 ms, FA : 12°,FoV:256 × 256 mm^2, matrix size:256 × 256,1mm thick, number of slices:192; the scanning time was 4 min 15 s	ICV
Cognitive decline	Kübler; 2022	Increased NBM volume correlated with improved cognitive outcome measured by MMSE or DemTect scores.	NBM volume by VBM using CAT12	1.5T or 3T	NR	the number of cognitive domains affected pre-operatively and disease severity
Psychiatric complications	Bourne; 2012	1).Pts with postoperative confusion had a larger minimum width of the lateral ventricles than controls.<br>2).Pts discharged to a higher level of care and those with any complications had larger maximum and minimum ventricular widths than controls.<br>3). Larger ventricles did not correlate with higher occurrence of ventricular penetration。	maximum and minimum lateral ventricular widths were measured on preoperative axial MRIs	NR	/	For each subject with one of the end points, an age-, sex-, diagnosis-, and DBS target-matched control was chosen from the same study cohort.

Psychiatric complications	Hrabovsky; 2017	1).The duration of PD motor complications, L-DOPA equivalent dose, DSR Mattis,third ventricle length and width did not predict psotoperative mental alteration.<br>2).The incidence of postoperative mental alteration with intermammillary distance > 8 mm was 60%.	third ventricular length (by AC-PC distance) and width (by intermammillary distance)	NR	/	NR
Psychiatric complications	Tanaka; 2018	1).Age and total WM volume correlated with the duration of POD.<br>2).WM was reduced in the temporal stem, and the reduction in volume correlated with the duration of POD.<br>3).Gray matter atrophy did not correlate with POD.	brain volume using VBM by SPM12	1.5T or 3T	1.5T Philips Intera system:TR/TE:5450/2650 ms; FA:10°; matrix:512×512; slices:170; 2 mm thick; voxel size: 0.586×0.586×1mm^3;<br>3T GE Signa EXCITE system: TR/TE:8690/1920 ms; FA:18°; matrix: 512×512; slices: 248; 1 mm thick; voxel size:0.43×0.43×0.698 mm^3.	NR
Psychiatric complications	Wang;2019	UPDRS III, nonmotor symptoms scale for PD, PD sleep scale, preoperative length of stay and preoperative brain atrophy correlated with the occurrence of POD after DBS in the multivariate analysis.	visual inspection	NR	NR	NR

Psychiatric complications	Radziunas;2020	1).Differences of cortical thickness in 13 gyruses on the right hemisphere (superior frontal, caudal middle frontal, pars triangularis and opercularis, temporal lobe, superior and inferior parietal,supramarginal) and in 7 gyruses on the left hemisphere (caudal middle frontal, inferior and middle temporal, pre and postcentral,superior parietal and supramarginal) were observed in pts with and without postoperative neuropsychiatric complications.<br>2).Reduced white matter volume in the left caudal middle frontal area were observed in pts with postoperative neuropsychiatric complications compared to pts without.<br>2).Volumes of subcortical structures did not correlate with postoperative neuropsychiatric complications.	cortical thickness by freesurfer,brain volume using VBM by freesurfer	1.5T	T2W: TR/TE: 4740/104 ms; 2.0 mm thick; FoV: 250 (192 × 256); concatenation:2, FA:120°; T2W/spcp2/iso: TR/TE：3200/376 ms; 1.0 mm thick; FoV 260 (256 × 256); concatenation 1; T1W/mpr/p2/iso: TR/TE 1900/3.35 ms; 1.0 mm thick; FoV:260 (192 × 256); concatenation:1, FA:15°	Eighteen healthy volunteers matched by age, gender and MMSE were selected as a control group

PDD = PD patients with dementia; PDnD = PD patients without dementia; HV = Hippocampal volume; POD = postoperative delirium; STN= subthalamic nucleus; WML = white matter lesions; ICV = intracranial volume; UPDRS = Unified Parkinson's Disease Rating Scale; CVLT-II = The California verbal learning test-II; HAMD = Hamilton Depression Scale; MMSE = Mini Mental Status Examination; MDRS = Mattis Dementia Rating Scale; MOCA = Montreal Cognitive Assessment; LED = L-DOPA equivalent dose; TR = repetition time; TE = echo time; TI = inversion time; FoV = field of view; FA = flip angle; BW =bandwidth; pts = patients;yr = year ; mo = month; d = day; min = minute; ms = millisecond; NR = not recorded.

Supplementary Table 3. DBS procedures in the studies included

topic	author; year	anesthesia	head frame	guiding techniques	electrode model
Motor outcome; quality of life	Bonneville; 2005	local	NR	intraoperative microelectrode recording and stimulation	3389 Medtronic
Motor outcome; quality of life	Hamasaki; 2010	NR	NR	intraoperative semi-microelectrode recording	3389 Medtronic
Motor outcome; axial symptoms	Price; 2011	NR	NR	intraoperative microelectrode recording and stimulation	NR
Motor outcome	Muthuraman; 2017	NR	stereotactic ring (Zamorano-Dujovny open ceramic version)	intraoperative microelectrode recording	3389 Medtronic
Motor outcome	Younce; 2019	local	Leksell stereotactic head frame	intraoperative microelectrode recording and stimulation	3389 Medtronic
Motor outcome; quality of life	Frizon; 2020	local	Leksell G Model frame	intraoperative imaging and microelectrode recording	3389 Medtronic
Motor outcome; levodopa response	Hamed; 2020	general/local	Leksell stereotactic head frame	intraoperative microelectrode recording or intraoperative CT imaging	3387 Medtronic
Motor outcome	Yim; 2020	general/local	Leksell Frame G	direct preoperative MRI targetting	3389 Medtronic
quality of life; motor outcome; depression; cognitive decline	Lu; 2021	NR	NR	NR	NR
Motor outcome	Chen; 2022	NR	Leksell G frame system	intraoperative microelectrode recording and stimulation	3389 Medtronic or L301 PINS
Motor outcome; levodopa response	Jergas; 2022	NR	NR	NR	NR
Fall and FOG	Karachi; 2019	NR	NR	intraoperative microelectrode recording and stimulation	3389 Medtronic
Gait variability	Wilkins;2020	local	functional frameless stereotactic technique	intraoperative microelectrode recording and stimulation	3389 Medtronic
Cognitive decline	Aybek; 2009	NR	NR	NR	NR
Cognitive decline	Geevarghese;2016		NR	NR	NR

Cognitive decline	Blume; 2017	NR	NR	NR	NR
Cognitive decline	Puy; 2018	general	Leksell stereotactic head frame	NR	NR
Cognitive decline	Planche; 2018	NR	NR	anatomic mapping and intraoperative microelectrode recordings	NR
Cognitive decline	Weinkle; 2018	local	NR	intraoperative microelectrode recording and stimulation	3389 Medtronic
Cognitive decline	Kübler; 2022	general/local	NR	NR	NR
Psychiatric complications	Bourne; 2012	NR	NR	NR	NR
Psychiatric complications	Hrabovsky; 2017	local	frame-based stereotaxy(ceramic MRI-compatible frame Zamorano Dujovny stereotactic system)	intraoperative microelectrode recording and stimulation	3389 Medtronic
Psychiatric complications	Tanaka; 2018	general	NR	NR	3389 or 3387 Medtronic
Psychiatric complications	Wang;2019	local	the Leksell U-G stereotactic instrument	intraoperative microelectrode recording and stimulation	3387 Medtronic or L301 PINS
Psychiatric complications	Radziunas;2020	general	Leksell G frame	intraoperative CT scan	NR

NR = not recorded.
